# Determinants of unhealthy living by gender, age group, and chronic health conditions across districts in Korea using the 2010-2017 Community Health Surveys

**DOI:** 10.4178/epih.e2024014

**Published:** 2024-01-04

**Authors:** Thi Tra Bui, Thi Huyen Trang Nguyen, Jinhee Lee, Sun Young Kim, Jin-Kyoung Oh

**Affiliations:** 1 Department of Cancer Control and Population Health, National Cancer Center Graduate School of Cancer Science and Policy, Goyang, Korea; 2 Division of Cancer Prevention, National Cancer Control Institute, National Cancer Center, Goyang, Korea; 3 Department of Cancer AI and Digital Health, National Cancer Control Institute, National Cancer Center, Goyang, Korea

**Keywords:** Chronic disease, Health inequities, Health surveys, Life style, Republic of Korea

## Abstract

**OBJECTIVES:**

We investigated the prevalence and determinants of unhealthy living by gender, age, and comorbidities across Korean districts.

**METHODS:**

For 806,246 men and 923,260 women from 245 districts who participated in the 2010-2017 Korean Community Health Surveys, risk scores were calculated based on obesity, physical inactivity, smoking, and high-risk alcohol consumption, each scored from 0 (lowest risk) to 2 (highest risk). A risk score ≥4 was defined as indicating unhealthy living, and weighted proportions were calculated for each district. Using multivariate regression, an ecological model including community socioeconomic, interpersonal, and neighborhood factors was examined by gender, age, and comorbidities.

**RESULTS:**

The mean age-standardized rate of unhealthy living was 24.05% for men and 4.91% for women (coefficients of variation, 13.94% and 29.51%, respectively). Individuals with chronic diseases more frequently exhibited unhealthy lifestyles. Unhealthy lifestyles were associated with educational attainment (β-coefficients: men, -0.21; women, -0.15), high household income (β=0.08 and 0.03, respectively), pub density (β=0.52 and 0.22, respectively), and fast-food outlet density (β=2.81 and 1.63, respectively). Negative associations were observed with manual labor, social activity participation, and hospital bed density. Unhealthy living was positively associated with living alone among women and with being unemployed among middle-aged men. Access to parks was negatively associated with unhealthy living among young men and women. The ecological model explained 32% of regional variation in men and 41% in women.

**CONCLUSIONS:**

Improving the neighborhood built and socioeconomic environment may reduce regional disparities in lifestyle behaviors; however, the impacts may vary according to socio-demographic traits and comorbidities.

## GRAPHICAL ABSTRACT

[Fig f2-epih-46-e2024014]

## Key Message

• District prevalence rates of unhealthy living were higher among men than women and decreased with advancing age.

• Efforts to reduce regional disparities in lifestyle behaviors could benefit from enhancements to the neighborhood environment and the socioeconomic status of the area.

• The effectiveness of such improvements may vary based on socio-demographic characteristics and health conditions.

## INTRODUCTION

Behavioral risk factors, including high-risk alcohol consumption, smoking, obesity, physical inactivity, and poor diet, impose an enormous burden on global health [1,2]. Unhealthy behaviors have been estimated to contribute to 60% of the causes of chronic diseases [3], leading to over 23 million deaths and constituting 36.5% of disability-adjusted life years in 2017 [4]. Beyond physical health, behavioral risk factors are associated with poor mental well-being [5,6]. Importantly, these risk factors often cluster within individuals and may interact, amplifying their impact on health [7,8]. As such, addressing clustered unhealthy lifestyle behaviors is crucial in the prevention and management of chronic diseases [1,3]. While people residing in the same area tend to exhibit similar lifestyle patterns, lifestyle risk factors may be unevenly distributed across regions, contributing to health disparities [3,9]. Consequently, understanding the clustering of unhealthy lifestyles through a community-based approach is vital for informing public health policies, especially those focused on community health.

Furthermore, studies have shown that unhealthy lifestyle patterns are associated with socio-demographic characteristics and health conditions [10-12]. Previous research has indicated that certain behavioral risk factors are more prevalent in women, while others are more common in men; moreover, these gender differences often vary with age [13]. A direct comparison of the crude prevalence of clustered unhealthy lifestyles in patients with chronic diseases to that of the general population could yield a skewed interpretation, as chronic diseases are more frequently observed in older individuals [14]. To address age and gender differences, some studies have employed standardized estimates or adjusted for those factors using statistical models [9,15]. However, these approaches may obscure the full nuances of factors associated with health practices in men and women across age groups, which could be better understood through a stratified analysis. In addition, heterogeneity across studies, such as the definitions of clustered unhealthy lifestyles and the covariates examined, can impede the synthesis of findings from multiple studies, which is necessary to gain a broader understanding of health lifestyles among diverse subpopulations [12,15,16].

As such, it is important to explore the clustering of unhealthy lifestyles at the regional level, while considering socio-demographic traits and health conditions. In Korea, addressing regional health disparities has been recognized as a goal for achieving health equity, as outlined in the National Health Plan 2020 [17]. The present study was designed to examine the prevalence and determinants of clustered unhealthy lifestyles across 245 districts in Korea, stratified by gender, age group, and major chronic health conditions, including depression, self-rated poor health, hypertension, diabetes, and arthritis. For this purpose, we employed the composite measure of lifestyle risk score, calculated as the sum of individual risk scores for each factor based on the level of risk [18]. The value of this indicator has been demonstrated in various studies at the individual level [18]; however, it has not yet been applied in an ecological investigation. In our research, the lifestyle risk score was derived from 4 key risk factors for chronic diseases: cigarette smoking, alcohol consumption, obesity, and physical inactivity. At the community level, we estimated the rate of unhealthy living as the prevalence of clustered unhealthy behaviors. To enable comparison across subpopulations, we utilized the same ecological model for all subgroups, applying age-standardized rates of unhealthy living.

## MATERIALS AND METHODS

### Data source and study participants

The Korea Community Health Survey (KCHS) is a nationwide community-based survey conducted annually by the Korea Disease Control and Prevention Agency since 2008. Each district includes approximately 900 participants aged 19 years or older, amounting to a total of about 220,000 participants per year. To ensure the representativeness and reliability of the data, random sampling methods are utilized. The KCHS is carried out by trained interviewers who visit the households of the selected participants. The data collected by the KCHS encompass socioeconomic characteristics, health practices and behaviors, utilization of medical services, and common health issues. This information is gathered through face-to-face interviews using a structured questionnaire. Further details regarding the KCHS can be found in previously published research [19].

To obtain more precise community-level estimates, we pooled the data from 1,830,260 individuals who participated in the 2010- 2017 KCHS. The units of analysis were 245 districts, which were reclassified to reflect changes in administrative divisions over the study period [20]. After excluding 100,754 individuals due to incomplete data for height, weight, physical activity, smoking, or alcohol consumption, our study population consisted of 1,729,506 participants, comprising 806,246 men and 923,260 women. As not every common health outcome in the KCHS was recorded annually, we focused on 5 subgroups corresponding to specific comorbidities [14,19]. The subgroup of participants with poor subjective health included individuals who self-reported their health status as “poor” or “very poor”. The depression subgroup encompassed those who had experienced depressive symptoms for more than 2 weeks in the preceding year. The hypertension subgroup was made up of people aged 30 years or older who had received a hypertension diagnosis from a physician. Similarly, the diabetes subgroup included individuals aged 30 years or older diagnosed with diabetes, and the arthritis subgroup consisted of those aged 50 years or older with an arthritis diagnosis. Age restrictions for these subgroups were based on the KCHS Data Usage Guidelines (http://chs.cdc.go.kr). The general characteristics of the study participants are detailed in Supplementary Material 1.

Information regarding covariates was estimated from the individual-level KCHS data or sourced from the Korean Statistical Information Service website (https://kosis.kr) (Table 1).

### Measurements

#### Dependent variable

Lifestyle indices can vary across studies, but they typically include factors such as smoking, alcohol consumption, physical inactivity, excess weight, and diet [18]. Due to a lack of dietary data, the lifestyle risk score used in this study was composed of 4 unhealthy characteristics or practices: obesity, physical inactivity, smoking, and high-risk alcohol consumption. Prior research has often characterized integrated unhealthy or healthy lifestyle patterns based on the number of lifestyle risk factors or healthy behaviors [11,15,21]. Considering the level of risk associated with each factor, a 3-tiered scoring system was applied in the present study, with scores ranging from 0 (indicating the lowest risk) to 2 (representing the highest risk) [22-24] (Supplementary Material 2). For each district, the prevalence of unhealthy living was estimated as the weighted proportion of participants with a lifestyle risk score of 4 or higher. This calculation was stratified by gender, age group, and comorbid conditions. Age-standardized rates of unhealthy living were employed in regression analyses, apart from the age group-stratified analyses, which used age-specific crude rates. The standard population for these calculations was the 2014 Korean population.

#### Independent variables

We employed an ecological model that incorporated community socioeconomic factors, community interpersonal factors, and community neighborhood factors (Table 1). The community socioeconomic factors included rates of living alone, receipt of basic living support, high equivalized household income, high education level, unemployment, and manual labor. We hypothesized that districts with higher rates of favorable socioeconomic conditions would display lower rates of unhealthy living. The community interpersonal factors examined included the rates of religious and social participation. We anticipated that greater participation in these activities would be associated with lower unhealthy living rates. The community neighborhood factors encompassed the density of pubs, fast-food outlets, and cigarette retailers per 1,000 residents, along with the per capita park area, the number of hospital beds per 1,000 residents, the health check-up participation rate, and the rate of unmet medical needs. We expected that easier access to alcohol, fast food, and tobacco would promote unhealthy living habits. In contrast, the availability of healthcare services and facilities was hypothesized to contribute to a lower rate of unhealthy living by encouraging participation in health promotion programs. Multiple studies have demonstrated associations between these factors and lifestyle practices [9,12,15,21]. The model included age-standardized rates for covariates.

### Statistical analysis

Means, standard deviations (SDs), and minimum and maximum values were calculated for both the risk score and the rates of clustered unhealthy lifestyle behaviors at the community level, using several risk score thresholds (≥ 2, ≥ 4, and ≥ 6). A score of 4 or higher was determined to be the most suitable cut-off for defining unhealthy living in the regression analyses. Descriptive statistics were then provided for the estimated prevalence of unhealthy living, broken down by this risk score categorization and further stratified by gender, age group, and comorbidities. To account for the complex sampling design of the KCHS, weights were applied using PROC SURVEY procedures in SAS version 9.4 (SAS Institute Inc., Cary, NC, USA). Regional variations in unhealthy living rates were assessed based on the coefficient of variation (CV), which represents the ratio of the SD to the mean, expressed as a percentage. Multiple linear regression analysis was used to identify determinants of unhealthy living by gender and age group in the general population, including those with and without comorbidities, as well as among subgroups with major chronic health conditions. The results of these analyses were reported in terms of regression coefficients and the explanatory power of the models, as indicated by R-squared (R^2^) values. The contribution of each covariate to the regional variation in unhealthy living rate was evaluated by subtracting the R^2^ value of a reduced model that excluded the covariate in question from that of the full model, which included all covariates. To support the main findings, regression analyses were conducted for individual risk factors and types of residential areas, categorized by administrative districts: *gu* for metropolitan, *si* for urban, and *gun* for rural areas [20].

### Ethics statement

The Korean Community Health Survey protocols received approval from the Research Ethics Review Committee of the Korea Centers for Disease Control and Prevention (2010-02CON-22-P; 2012-07CON-01-2C; 2014-08EXP-09-4C-A; 2016-10-01-P-A). The present study was granted an exemption from review by the Institutional Review Board of the National Cancer Center, Korea (NCC2023-0114).

## RESULTS

### Community-level prevalence of unhealthy living

**Figure 1.**
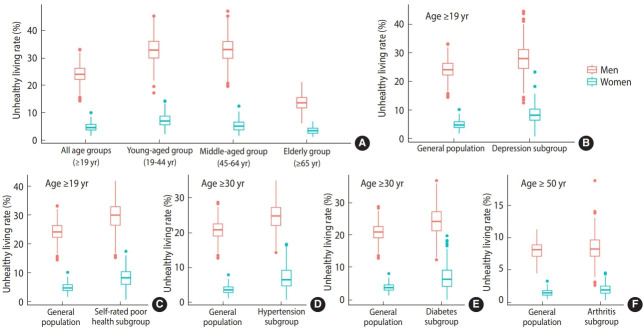
Community-level rates of unhealthy living, stratified by gender, (A) age group, and chronic health conditions, (B) depression, (C) self-rated poor health, (D) hypertension, (E) diabetes, and (F) arthritis.

The mean risk score was 2.68 (CV= 8.25%) for men and 1.80 (CV= 10.59%) for women across the 245 districts (Table 2). The mean age-standardized prevalence of a risk score of 4 or higher was 24.05% among men and 4.91% among women, with variation among districts (CV= 13.94% for men, 29.51% for women). The prevalence of clustered unhealthy lifestyle behaviors varied substantially according to different categorizations based on the risk score. For instance, up to 61.68% of men (CV= 5.61%) and 54.95% of women (CV= 10.55%) had a risk score of 2 or higher, whereas 4.91% of men (CV= 23.82%) and 0.36% of women (CV= 51.93%) had a risk score of 6 or higher. Unhealthy living was considered to be indicated by a risk score of 4 or above. In the general population, substantial gender differences in the rates of unhealthy living were observed across all age groups, with men more prone to engage in unhealthy behaviors than women (Figure 1 and Table 2). Regarding age, the prevalence of unhealthy living reached its peak in middle-aged men (defined as men 45-64 years old) and in younger women (defined as those 19-44 years old). Compared to rural areas, urban and metropolitan regions displayed higher rates of unhealthy living among both men and women. However, these disparities in unhealthy living rates decreased after age standardization. The variation in rate across districts was more pronounced for women than for men. Relative to the general population, the age-standardized prevalence of unhealthy living was elevated in subgroups with chronic health conditions, with the exception of those with arthritis.

### Community-level determinants of unhealthy living

Table 3 presents the results of regression analyses identifying factors associated with unhealthy living at the community level, differentiated by gender and age group. Across genders, the prevalence of unhealthy living was negatively associated with educational attainment and positively associated with higher household income and the density of pubs and fast-food outlets. Additionally, inverse associations were observed with the prevalence of individuals engaged in manual labor, social activities, and the density of hospital beds. Among women, a positive association was found between unhealthy living prevalence and the proportion of individuals living alone. Our model accounted for 32% of the regional variation in unhealthy living rates among men and 41% among women. For both men and women, community neighborhood factors contributed the greatest explanatory power.

Across age groups, the rate of unhealthy living was consistently and significantly associated with educational attainment for both men and women, with the density of pubs for men, and with the density of fast-food outlets for women (Table 3). Certain determinants were identified for specific age groups. For example, for both men and women within the youngest age group, a negative association was observed between unhealthy living and access to parks. Additionally, for men and women aged 45 years and older, unhealthy living was negatively associated with the rates of health check-ups and unmet medical needs. In contrast, a positive association with the unemployment rate was noted among middle-aged men. Notably, the rate of participation in religious activities displayed a negative association with unhealthy living among young men but a positive association among middle-aged women. The explanatory power of the model was highest for the middle-aged subgroups (41% for men and 44% for women).

The potential determinants of unhealthy living appeared to differ across subgroups categorized by chronic disease status (Table 4). However, a consistent positive association with the density of fast-food outlets was observed across disease and gender subgroups, with the exception of men with arthritis and women with depression. For women, educational attainment and per capita park area remained important protective determinants. Among men with hypertension or diabetes, a positive relationship was noted between unhealthy living and the rate of health check-ups. The rate of unemployment was positively associated with unhealthy living in the arthritis subgroup regardless of gender. Our model was able to moderately explain the regional differences in unhealthy living within the major chronic health condition subgroups (R^2^, 10-23% in men and 12-26% in women).

## DISCUSSION

In this study, we examined the prevalence and determinants of unhealthy living at the community level, differentiated by gender, age group, and the presence of major chronic health conditions. The rate of unhealthy living was markedly higher among men than among women and decreased with advancing age. Individuals with depression, self-perceived poor health, hypertension, or diabetes were particularly prone to engage in multiple unhealthy behaviors. Our ecological model explained 32% and 41% of the variation in unhealthy living among healthy men and women, respectively, across districts. However, it only modestly accounted for the variation among subgroups categorized by chronic disease status. The determinants of unhealthy living appeared to vary based on gender, age group, and the presence of comorbidities. Educational attainment and the availability of pubs and fast-food outlets emerged as particularly important determinants, supporting our hypotheses.

Several studies have demonstrated gender disparities in unhealthy lifestyle behaviors [11,25]. Men may tend to engage in risky substance use, whereas women are generally less likely to smoke or consume alcohol due to societal stigma, particularly in Asian cultures [13,25,26]. Conversely, women are often less actively involved in physical activity, as family responsibilities often take precedence and present practical challenges [25,27]. Furthermore, obesity rates among Korean women exceed those of Korean men [28]. Thus, it was anticipated that a markedly higher rate of unhealthy living—an integrated indicator—among men would primarily be attributed to gender differences in tobacco and alcohol use. Both elderly men and elderly women often quit or reduce their smoking and drinking habits as they become more aware of the adverse health impacts associated with these behaviors [29]. Separately, social expectations of being “a good wife and wise mother” may deter middle-aged women from participating in these unhealthy practices after marriage [26]. A prior study indicated that health behaviors among individuals with chronic diseases were not better, and were in some cases worse, than those observed in the general population [14]. In our research, we distinctly noted a higher age-standardized rate of unhealthy living among subgroups with chronic health conditions, arthritis patients being the exception.

Regarding community socioeconomic factors, unhealthy living was negatively associated with educational attainment across all age groups and genders. Educational attainment was the largest contributor to regional disparities in unhealthy living among the various factors examined. Notably, the explanatory power of educational attainment alone (R^2^ = 0.07 in men, 0.13 in women) was considerably smaller than that of the full model (R^2^ = 0.32 in men, 0.41 in women), which accounted for other covariates that were controlled for and to some extent correlated with each other. When stratified by residential area, educational attainment continued to be a key determinant of the rate of unhealthy living in metropolitan areas (for both men and women) and in urban areas (for women) (Supplementary Material 3). A previous study utilizing the 2010 KCHS data indicated that low educational attainment was linked to a lower prevalence of healthy lifestyles, corroborating our findings [11]. More highly educated individuals may make healthier choices due to greater financial capacity and health literacy [30]. Unhealthy behaviors such as smoking are potentially associated with low self-control, an attraction to risk, and a preference for immediate gratification over long-term well-being, which may be more common among those with lower educational attainment [31]. In the subgroups of participants with chronic health conditions, this association was not evident among men, while it was clearly present among women. Analysis of specific lifestyle practices in men participants revealed that educational attainment was negatively associated with smoking but positively associated with obesity, physical inactivity, and high-risk alcohol consumption, although these associations were not statistically significant (Supplementary Material 4). Evidence suggests that more highly educated men tend to have sedentary jobs with long working hours or to engage in high-risk alcohol consumption as part of workplace culture. Moreover, compared to women, men are more prone to underestimating their body size [32].

Regarding community interpersonal factors, a negative association was observed between unhealthy living and participation in social activities among young and middle-aged women, supporting our hypotheses. Social connections may encourage healthy behaviors through the fostering of positive psychological states, yet they may also promote undesirable behaviors, such as smoking or alcohol consumption [33]. A previous study suggested that the specific activities engaged in may hold more importance for health and well-being than the general nature of social involvement [34]. Future research should employ specific measures of social activity to confirm this finding. In the present study, we noted a negative association between unhealthy living and participation in religious activities among young men, but we found a positive relationship among middle-aged women. Engagement in religious activities may promote healthy habits at the community level by disseminating health information and establishing social norms that support healthy living and discourage risky behaviors [21]. At the individual level, religious activities may contribute to stress reduction and enhanced resilience [35]. Many religions also impose restrictions on substance use, such as smoking and heavy drinking [35]. However, religious moral perspectives may exhibit less social stigma towards individuals with obesity, rendering religious organizations a comforting and inclusive environment [36]. A study among postmenopausal women identified links between regular worship attendance and both non-smoking and moderate alcohol consumption, but it did not reveal a significant association with regular exercise or a diet low in saturated fat and calories [37].

In examining community neighborhood factors, we observed a negative association between unhealthy living and park area per capita among young adults regardless of gender, as well as among subgroups of women with chronic diseases. Green spaces appear to offer natural benefits, potentially aiding in healing and providing a buffer against depression, air pollution, noise, and heat waves [38]. The availability of green spaces not only encourages physical activity [12] but also may support the cessation of other risky behaviors through neurobiological pathways, such as enhanced self-efficacy and a strengthened sense of control [35]. Previous research indicated that physical activity rates were comparatively high in men and tended to decrease with age [39]. This trend could reflect the alignment with both aging processes and cultural expectations across genders [39]. A negative association was also identified between unhealthy living and the density of hospital beds for women of all ages, as well as for chronic disease subgroups. In Korea, the concentration of medical beds in urban centers [40] suggests that regions with sparse population may experience relatively poor access to medical services. This is supported by previous research indicating that women in smaller cities are less inclined to adopt healthy behaviors [12], which aligns with our results. The inverse association between unhealthy living and the rate of health check-ups in middle-aged and older men could be linked to heightened health awareness, which often comes with advancing age [41]. In contrast to findings from individual-level research [16], we identified a negative association between unhealthy living and unmet medical needs in middle-aged and older women. Women in rural areas were more likely to experience unmet healthcare needs than those in other settings [42]. Nevertheless, studies have shown that elderly rural residents tend to exhibit comparatively healthy behaviors, such as being more physically active and having lower obesity rates [43]. Women residing in areas of high socioeconomic status, by contrast, were more prone to smoking, a trend that might be explained by the concept of relative deprivation [44].

This study also identified several community-level factors that were positively associated with unhealthy living. Among middle-aged women, unhealthy living was linked to a higher prevalence of residing alone. Living alone, as opposed to cohabiting, has been found to be associated with worse physical and mental health, greater social isolation, and lower socioeconomic status [45]. To manage induced stress and depression, individuals living alone might turn to smoking, drinking, inactivity, or overeating as forms of relaxation [31,45]. Additionally, it is possible that unmarried, widowed, or divorced women may become habitual smokers due to the absence of perceived social constraints or peer support to encourage quitting [31]. Contrary to our hypothesis, districts with greater rates of high equivalized household income tended to exhibit higher rates of unhealthy living. Stratified by residential area, this pattern was only observed in rural districts, which was not statistically significant (Supplementary Material 3). One previous study indicated that high financial independence was not associated with healthy lifestyle, leaving the causal link between better financial autonomy in a community and the presence of infrastructure promoting healthy living unclear [15]. This underscores the importance of resource distribution at the community level. We also observed a positive association between unemployment and unhealthy living among middle-aged men and arthritis patients regardless of gender. Unemployment can lead to financial stress and depression [46], conditions that may be particularly likely to manifest during middle age—a period typically characterized by peak economic productivity—or among arthritis patients due to limited mobility. Unemployment may prompt individuals to seek immediate gratification through behaviors like smoking or alcohol consumption, without fully considering the long-term health implications [47]. Regarding community neighborhood factors, a higher rate of unhealthy living was consistently associated with a greater density of pubs and fast-food outlets across the general population and among chronic disease subgroups, regardless of gender. This finding aligns with previous research [9,15,31]. The concentrations of these establishments helped account for geographic disparities in unhealthy living rates in both urban and rural areas, though not in metropolitan regions (Supplementary Material 3). Previous research has demonstrated that communities with a greater density of alcohol outlets face a higher risk of issues related to excessive drinking [48]. Since these outlets have been found to be more dense in deprived areas, where excessive drinkers are more susceptible to alcohol-related harm compared to those in more advantaged areas, these findings suggest that policy interventions to limit alcohol availability may help to reduce health disparities [48,49]. A Dutch study highlighted the link between the proximity of fast-food outlets within 500 m and increased body mass index, with this association being stronger in rural areas than urban ones [50]. This finding may be attributed to the scarcity and limited operating hours of healthy food options, such as supermarkets, in rural locations [50].

Based on our findings, in addition to the improvement of socioeconomic conditions in the area, we suggest that the enhancement of neighborhood environments may contribute to promoting healthy lifestyles. Policies limiting the availability of alcohol should be reinforced, and nutritious food should be made more readily available. Primary care has the potential to offer health promotion services or programs, including nutrition counseling, smoking cessation services, or interventions to increase physical activity. Thus, strengthening the primary care system can aid in addressing regional health disparities. Proactive strategies are necessary to promote healthy living among individuals with chronic diseases.

This study had several strengths. First, we employed the KCHS, a large-scale nationwide population-based survey, which enabled us to generate representative indicators at the community level. Second, we utilized lifestyle risk score as a singular indicator of the combination of unhealthy behaviors, providing us with a broader perspective on lifestyle practices across regions. Additionally, our ecological model incorporated multiple covariates that were either age-standardized or specified according to age, gender, and comorbidities. Regarding limitations, the present ecological analysis examined data across communities, which may yield results that differ from those obtained through individual-level investigation. We also assumed that several community neighborhood factors uniformly impacted all individuals within the subpopulations. Furthermore, as this study relied on survey data, we could not establish a causal relationship between unhealthy lifestyles and the examined covariates. Additionally, the use of self-reported data in this study may have introduced bias due to underreporting or over-reporting.

In summary, the rates of unhealthy living at the community level were higher among men than women and decreased with advancing age. Efforts to reduce regional disparities in lifestyle behaviors could benefit from enhancements to the neighborhood environment and the socioeconomic status of the area. The effectiveness of such improvements may vary based on socio-demographic characteristics and health conditions.

## Figures and Tables

**Figure f2-epih-46-e2024014:**
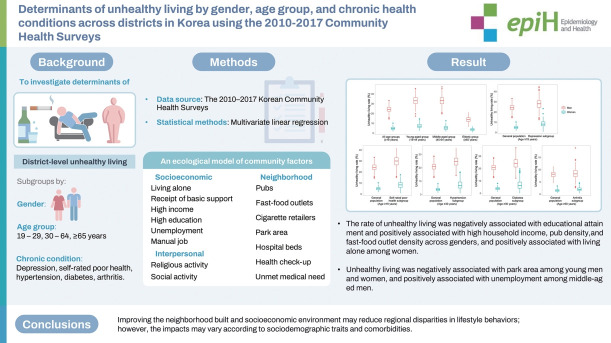


**Table 1.** Variables and data sources

Variables		Definition	Data source	Year
Dependent variable	Unhealthy living^1^	Weighted proportion of participants with a lifestyle risk score of 4 or higher; lifestyle risk score includes obesity, physical inactivity, smoking, and high-risk alcohol consumption, with each scored between 0 (lowest risk) and 2 (highest risk)	KCHS	2010-2017
Independent variables				
Community socioeconomic factors^1^	Living alone	Weighted proportion of participants living alone (%)	KCHS	2014
	Receipt of basic support	Weighted proportion of participants receiving basic living support (%)	KCHS	2014
	High income	Weighted proportion of participants with equivalized household incomes higher than the median of equivalized household income among participants (%)	KCHS	2013
	High education	Weighted proportion of participants who graduated from university or higher (%)	KCHS	2014
	Unemployment	Weighted proportion of participants aged under 65 yr reporting unemployment (%)	KCHS	2014
	Manual job	Weighted proportion of participants aged under 65 yr with jobs involving manual labor (%)	KCHS	2014
Community interpersonal factors	Religious activity^1^	Weighted proportion of participants who participate in religious activities at least once a month (%)	KCHS	2015
	Social activity^1^	Weighted proportion of participants who participate in social activities (class gatherings, alumni associations, events at senior citizen centers, etc.) at least once a month (%)	KCHS	2015
Community neighborhood factors	Pubs	No. of pubs per 1,000 people	KOSIS	2014
	Fast-food outlets	No. of fast-food outlets (pizza, hamburger, sandwich, or chicken restaurants) per 1,000 people	KOSIS	2014
	Cigarette retailers	No. of cigarette retailers per 1,000 people	KOSIS	2014
	Park area	Park area per person (m^2^)	KOSIS	2014
	Hospital beds	No. of beds in medical hospitals per 1,000 people	KOSIS	2014
	Health check-ups^1^	Weighted proportion of participants who received health check-ups within the prior 2 yr (excluding cancer screening) (%)	KCHS	2014
	Unmet medical need^1^	Weighted proportion of participants reporting unmet medical need (not including dental care) in the prior year (%)	KCHS	2014

KCHS, Korea Community Health Survey; KOSIS, Korean Statistical Information Service.

1 Specific estimates were made by gender, age group, and chronic health condition subgroup.

**Table 2.** Descriptive statistics for community-level risk scores and rates of unhealthy living

Variables		Men				Women			
		Mean±SD	Min	Max	CV	Mean±SD	Min	Max	CV
Risk score	Overall	2.68±0.22	1.99	3.22	8.25	1.80±0.19	1.14	2.21	10.59
	Age group (yr)								
	Young (19-44)	2.81±0.21	2.10	3.31	7.57	1.87±0.17	1.35	2.25	9.04
	Middle age (45-64)	2.78±0.22	2.15	3.42	7.99	1.70±0.21	1.01	2.19	12.34
	Elderly (≥65)	2.10±0.20	1.42	2.56	9.55	1.86±0.21	1.04	2.23	11.50
	Crude rate of risk score ≥2	76.62±4.59	59.76	87.73	5.99	68.28±7.57	40.23	82.30	11.09
	ASR of risk score ≥2	61.68±3.46	49.78	70.45	5.61	54.95±5.80	34.11	65.32	10.55
	Crude rate of risk score ≥4	29.82±4.58	17.24	42.75	15.35	5.87±1.84	2.00	12.20	31.40
	ASR of risk score ≥4	24.05±3.35	14.45	33.21	13.94	4.91±1.45	1.72	10.16	29.51
	Crude rate of risk score ≥6	6.05±1.53	2.25	10.54	25.35	0.43±0.24	0.01	1.34	55.02
	ASR of risk score ≥6	4.91±1.17	1.78	8.35	23.82	0.36±0.19	0.01	1.14	51.93
Rate of unhealthy living (risk score ≥4)	General population (yr)								
	Crude rate ≥19	29.82±4.58	17.24	42.75	15.35	5.87±1.84	2.00	12.20	31.40
	ASR (≥19)	24.05±3.35	14.45	33.21	13.94	4.91±1.45	1.72	10.16	29.51
	Crude rate ≥30	31.04±4.80	17.34	44.88	15.45	5.50±1.77	1.77	11.71	32.20
	ASR (≥30)	20.63±2.82	12.73	28.68	13.66	3.80±1.16	1.28	8.01	30.58
	Crude rate ≥50	24.47±4.36	13.16	36.79	17.83	4.47±1.51	1.32	9.51	33.77
	ASR (≥50)	8.04±1.29	4.51	11.32	16.09	1.51±0.50	0.53	3.31	32.81
	Age group crude rate (yr)								
	Young (19-44)	33.00±4.72	17.37	45.40	14.30	7.40±2.25	2.37	14.41	30.41
	Middle age (45-64)	33.03±4.85	19.74	47.17	14.67	5.40±1.85	1.76	12.56	34.24
	Elderly (≥65)	13.73±2.98	6.30	21.32	21.73	3.64±1.33	0.86	6.99	36.39
	By area of residence (crude rate ≥19 yr)								
	Metropolitan	30.94±3.82	20.63	42.75	12.34	6.22±1.62	2.71	10.86	26.03
	Urban	31.03±4.20	18.34	39.42	13.54	6.45±1.82	2.14	12.20	28.19
	Rural	27.52±4.86	17.24	37.55	17.67	4.98±1.80	2.00	9.29	36.15
	By urban-rural classification (ASR ≥19 yr)								
	Metropolitan	24.20±3.10	15.77	33.21	12.82	5.00±1.31	2.14	8.67	26.21
	Urban	24.60±3.10	14.45	31.76	12.58	5.29±1.47	1.72	10.16	27.89
	Rural	23.43±3.75	14.70	31.78	16.01	4.52±1.51	2.05	8.28	33.45
	Depression subgroup (yr)								
	Crude prevalence ≥19	33.98±6.11	20.93	51.53	17.98	9.44±3.19	1.19	18.08	33.81
	ASR (≥19)	28.07±5.53	12.51	44.64	19.71	8.33±2.99	0.76	23.33	35.91
	Self-rated poor health subgroup (yr)								
	Crude prevalence ≥19	28.34±5.89	13.92	44.07	20.76	6.74±2.46	1.28	12.80	36.51
	ASR (≥19)	29.56±4.94	15.25	41.86	16.70	8.16±3.05	0.73	17.44	37.38
	Hypertension subgroup (yr)								
	Crude prevalence ≥30	28.53±5.47	14.40	45.91	19.18	6.33±2.19	1.58	11.90	34.55
	ASR (≥30)	24.56±3.68	14.34	34.85	14.99	7.25±3.41	0.92	16.72	47.01
	Diabetes subgroup (yr)								
	Crude prevalence ≥30	29.12±4.89	17.23	44.60	16.79	7.13±2.62	0.01	14.87	36.76
	ASR (≥30)	24.10±4.42	12.27	36.68	18.32	6.77±3.69	0.01	19.66	54.61
	Arthritis subgroup (yr)								
	Crude prevalence ≥50	20.21±5.58	4.52	49.16	27.63	5.30±1.97	1.32	10.69	37.23
	ASR (≥50)	8.33±2.13	2.66	18.86	25.56	2.01±0.79	0.39	4.57	39.22

SD, standard deviation; Min, minimum; Max, maximum; CV, coefficient of variation; ASR, age-standardized rate.

**Table 3.** Results of regression analyses examining determinants of unhealthy living rate by gender and age group in the general population

Variables	Age (yr)							
	Total		19-44		45-64		≥65	
	β^1^	R^2^	β^2^	R^2^	β^2^	R^2^	β^2^	R^2^
Men	-	0.32	-	0.25	-	0.41	-	0.25
Community socioeconomic factors	-	0.09	-	0.06	-	0.13	-	0.04
Living alone	0.04	0.00	-0.02	0.00	0.11	0.00	0.05	0.00
Recipient of basic support	-0.13	0.00	-0.25	0.01	-0.43^***^	0.02	0.06	0.00
High income	0.08^**^	0.01	0.04	0.01	0.07^**^	0.01	0.05^**^	0.01
High education	-0.21^***^	0.07	-0.13^***^	0.02	-0.17^***^	0.07	-0.07^***^	0.02
Unemployment	0.04	0.00	0.04	0.00	0.20^**^	0.01	-	-
Manual job	-0.07	0.01	0.05	0.00	-0.09^**^	0.01	-	-
Community interpersonal factors	-	0.02	-	0.05	-	0.01	-	0.00
Religious activity	-0.06	0.01	-0.16^***^	0.04	-0.03	0.00	-0.01	0.00
Social activity	-0.06	0.01	-0.05	0.01	-0.06	0.01	-0.01	0.00
Community neighborhood factors	-	0.11	-	0.10	-	0.13	-	0.16
Pubs	0.52^***^	0.03	0.86^***^	0.04	0.56^**^	0.01	0.46^***^	0.03
Fast-food outlets	2.81^***^	0.03	2.07	0.01	3.75^***^	0.03	1.81^**^	0.02
Cigarette retailers	-2.66	0.00	-5.31	0.00	1.66	0.00	-1.40	0.00
Park area	-0.02	0.01	-0.03^**^	0.01	-0.02	0.01	-0.02	0.01
Hospital beds	-0.03	0.01	-0.04	0.00	-0.04	0.00	-0.04	0.01
Health check-ups	-0.07	0.01	0.04	0.00	-0.21^***^	0.05	-0.10^***^	0.05
Unmet medical need	0.05	0.00	0.06	0.00	-0.04	0.00	-0.02	0.00
Women	-	0.41	-	0.33	-	0.44	-	0.25
Community socioeconomic factors	-	0.15	-	0.06	-	0.17	-	0.07
Living alone	0.09^***^	0.03	0.02	0.00	0.05^**^	0.01	0.01	0.00
Recipient of basic support	-0.02	0.00	-0.08	0.00	-0.01	0.00	0.07^***^	0.02
High income	0.03^**^	0.01	0.01	0.00	0.02	0.01	0.02^**^	0.01
High education	-0.15^***^	0.13	-0.08^***^	0.05	-0.11^***^	0.12	-0.09^***^	0.04
Unemployment	-0.05	0.00	-0.01	0.00	0.01	0.00	-	-
Manual job	-0.06^**^	0.02	-0.01	0.00	-0.04^**^	0.02	-	-
Community interpersonal factors	-	0.02	-	0.04	-	0.05	-	0.01
Religious activity	0.01	0.00	-0.03	0.01	0.04^***^	0.03	0.01	0.01
Social activity	-0.03^***^	0.02	-0.04^***^	0.03	-0.04^***^	0.03	0.00	0.00
Community neighborhood factors	-	0.17	-	0.17	-	0.21	-	0.13
Pubs	0.22^***^	0.02	0.53^***^	0.06	0.21^**^	0.01	0.03	0.00
Fast-food outlets	1.63^***^	0.05	1.34^**^	0.01	2.41^***^	0.07	1.44^***^	0.05
Cigarette retailers	-1.90	0.00	-5.20	0.01	-0.79	0.00	0.20	0.00
Park area	-0.01	0.01	-0.02^***^	0.03	0.00	0.00	0.00	0.00
Hospital beds	-0.03^***^	0.03	-0.05^***^	0.04	-0.04^***^	0.03	-0.02^***^	0.02
Health check-ups	-0.02	0.00	-0.02	0.00	-0.02	0.00	-0.02	0.01
Unmet medical need	-0.02	0.00	0.00	0.00	-0.05^**^	0.01	-0.04^***^	0.03

β, regression coefficient; R^2^, R-squared value.

1 Estimated from regression models including age-standardized rates of unhealthy living as the dependent variable and age-standardized rates of covariates (except the densities of pubs, fast-food outlets, and cigarette retailers, as well as hospital beds per 1,000 people and park area per person, using crude estimates) as independent variables.

2 Estimated from regression models including age-specific crude rates of unhealthy living as the dependent variable and age-specific crude rates of covariates (except the densities of pubs, fast-food outlets, and cigarette retailers, as well as hospital beds per 1,000 people and park area per person, using crude rates for all age groups) as independent variables.

** p<0.01,

*** p<0.001.

**Table 4.** Results of regression analyses^1^ examining determinants of unhealthy living rate by gender within subgroups of individuals with specific chronic health conditions

Variables	Depression		Self-rated poor health		Hypertension		Diabetes		Arthritis	
	β	R^2^	β	R^2^	β	R^2^	β	R^2^	β	R^2^
Men	-	0.12	-	0.23	-	0.17	-	0.15	-	0.10
Community socioeconomic factors	-	0.03	-	0.05	-	0.03	-	0.03	-	0.06
Living alone	0.00	0.00	0.02	0.00	0.06	0.01	-0.06	0.00	-0.03	0.00
Recipient of basic support	0.01	0.00	-0.02	0.00	-0.20	0.01	-0.03	0.00	-0.06	0.01
High income	0.01	0.00	0.07^**^	0.02	0.02	0.00	0.07^**^	0.02	0.00	0.00
High education	-0.03	0.00	0.01	0.00	-0.05	0.01	0.01	0.00	-0.07	0.01
Unemployment	0.08	0.01	0.06	0.00	0.07	0.00	0.13	0.01	0.14^***^	0.03
Manual job	0.09^**^	0.02	-0.04	0.00	0.05	0.01	0.05	0.01	0.00	0.00
Community interpersonal factors	-	0.00	-	0.01	-	0.01	-	0.00	-	0.01
Religious activity	-0.04	0.00	-0.08	0.01	-0.06	0.01	-0.03	0.00	0.01	0.00
Social activity	0.02	0.00	0.01	0.00	-0.01	0.00	0.02	0.00	0.03	0.00
Community neighborhood factors	-	0.07	-	0.11	-	0.13	-	0.09	-	0.04
Pubs	0.32	0.00	0.50	0.01	0.49^**^	0.02	0.45	0.01	0.12	0.00
Fast-food outlets	4.70^***^	0.03	4.61^***^	0.04	2.55^**^	0.02	3.11^**^	0.02	0.70	0.00
Cigarette retailers	-11.95	0.01	-5.44	0.00	-7.59	0.01	-7.17	0.01	2.95	0.00
Park area	-0.02	0.01	-0.02	0.01	-0.02	0.01	-0.02	0.01	-0.01	0.01
Hospital beds	0.00	0.00	-0.08^**^	0.02	-0.04	0.01	0.03	0.00	-0.02	0.01
Health check-ups	0.01	0.00	0.04	0.00	0.10*	0.02	0.08^**^	0.02	-0.02	0.00
Unmet medical need	0.00	0.00	0.04	0.00	0.00	0.00	0.03	0.00	0.04	0.01
Women	-	0.16	-	0.18	-	0.12	-	0.17	-	0.26
Community socioeconomic factors	-	0.04	-	0.06	-	0.03	-	0.03	-	0.05
Living alone	0.02	0.00	0.08^***^	0.03	0.03	0.00	0.03	0.00	0.03	0.01
Recipient of basic support	-0.06	0.00	0.06	0.00	-0.05	0.00	0.04	0.00	0.01	0.00
High income	0.02	0.01	-0.01	0.00	0.02	0.00	0.01	0.00	0.00	0.00
High education	-0.07^***^	0.03	-0.02	0.00	-0.10^**^	0.02	-0.10^**^	0.02	-0.02	0.00
Unemployment	0.05	0.01	0.01	0.00	-0.11	0.01	-0.08	0.00	0.06^**^	0.01
Manual job	0.01	0.00	0.01	0.00	-0.08^**^	0.02	-0.01	0.00	-0.01	0.00
Community interpersonal factors	-	0.00	-	0.00	-	0.00	-	0.01	-	0.04
Religious activity	0.00	0.00	0.00	0.00	0.01	0.00	0.05	0.01	0.04^***^	0.03
Social activity	0.00	0.00	0.02	0.00	0.01	0.00	-0.03	0.00	-0.03^**^	0.02
Community neighborhood factors	-	0.09	-	0.09	-	0.09	-	0.13	-	0.11
Pubs	0.48^***^	0.03	0.26	0.01	0.30	0.01	0.18	0.00	0.06	0.01
Fast-food outlets	1.52	0.01	2.53^***^	0.03	2.74^***^	0.03	3.89^***^	0.05	0.77^***^	0.04
Cigarette retailers	-2.05	0.00	-6.95	0.01	-1.27	0.00	5.88	0.00	-0.93	0.00
Park area	-0.02^**^	0.02	-0.02^***^	0.03	-0.01	0.00	-0.02^**^	0.02	0.00	0.00
Hospital beds	-0.05^**^	0.02	-0.03	0.01	-0.05	0.01	-0.07^**^	0.02	-0.01	0.01
Health check-ups	0.01	0.00	0.02	0.00	0.05	0.01	0.04	0.01	-0.03	0.01
Unmet medical need	0.03	0.01	-0.02	0.00	0.04	0.00	-0.03	0.00	-0.02	0.01

β, regression coefficient; R^2^, R-squared value.

1 Estimated from regression models including age-standardized rates of unhealthy living as the dependent variable and age-standardized rates of covariates (except the densities of pubs, fast-food outlets, and cigarette retailers, as well as hospital beds per 1,000 people and park area per person, using crude estimates) as independent variables.

** p<0.01,

*** p<0.001.

## Data Availability

The data used in this study are available upon request via the Community Health Survey (CHS) website (http://chs.kdca.go.kr).

## Supplementary materials

Supplementary Material 1. General characteristics of study participants

Supplementary Material 2. Definition of unhealthy living at the individual level

Supplementary Material 3. Regression analysis results for the determinants of unhealthy living rates by gender, age group, and urbanity in the general population

Supplementary Material 4. Regression analysis results for the determinants of individual unhealthy lifestyle practice rates by gender in the subgroups of chronic health conditions
