# Supplementary material for: Determinants of unhealthy living by gender, age group, and chronic health conditions across districts in Korea using the 2010-2017 Community Health Surveys
Source: Epidemiol Health. 2024 Jan 4;46:e2024014. doi: 10.4178/epih.e2024014 (PMC11040218; doi:10.4178/epih.e2024014)
Supplement: Supplementary Material 1. — General characteristics of study participants [file epih-46-e2024014-Supplementary-1.docx]

Supplementary Material 1. General characteristics of study participants

| Characteristics | Men | | Women | |
| --- | --- | --- | --- | --- |
|  | N | % | N | % |
| General population, total | 806246 | 46.62 | 923260 | 53.38 |
| Young-aged group, 19-44 years | 303773 | 37.68 | 338618 | 36.68 |
| Middle-aged group, 45-64 years | 316199 | 39.22 | 359381 | 38.93 |
| Elderly, ≥65 years | 186274 | 23.10 | 225261 | 24.39 |
| Depression subgroup | 32348 | 4.01 | 68623 | 7.43 |
| Self-rated poor health subgroup | 129063 | 16.01 | 206957 | 22.42 |
| Hypertension subgroup | 186483 | 23.13 | 213724 | 23.15 |
| Diabetes subgroup | 80658 | 10.00 | 76659 | 8.30 |
| Arthritis subgroup | 42632 | 5.29 | 160239 | 17.36 |
