# Supplementary material for: Determinants of unhealthy living by gender, age group, and chronic health conditions across districts in Korea using the 2010-2017 Community Health Surveys
Source: Epidemiol Health. 2024 Jan 4;46:e2024014. doi: 10.4178/epih.e2024014 (PMC11040218; doi:10.4178/epih.e2024014)
Supplement: Supplementary Material 2. — Definition of unhealthy living at the individual level [file epih-46-e2024014-Supplementary-2.docx]

Supplementary Material 2. Definition of unhealthy living at the individual level

| Risk score | Obesity | Physical inactivity^a^ | Cigarette smoking | High-risk alcohol consumption^b^ | Unhealthy living |
| --- | --- | --- | --- | --- | --- |
| 0 | Non-obesity (BMI <25 kg/m^2^) | ≥3 times/week | Non-current smokers | Non-drinking or never high-risk consumption | An overall lifestyle risk score (as the sum of risk scores for obesity, physical inactivity, smoking, and high-risk alcohol consumption) ≥4 |
| 1 | Class I obesity (25 ≤ BMI <30 kg/m^2^) | 1-2 times/week | Light-to-moderate smokers (1-19 cigarettes/day for men, 1-9 cigarettes/day for women) | About once a month |  |
| 2 | Class II obesity (BMI ≥30 kg/m^2^) | No physical activity | Heavy smokers (≥20 cigarettes/day for men, ≥10 cigarettes/day for women) | About once a week or almost every day |  |

^a^ Do not engage in physical activity, such as vigorous exercise ≥20 minutes or moderate exercise ≥30 minutes.

^b^ ≥7 glasses for males and ≥5 glasses for females.
