# Supplementary material for: Determinants of unhealthy living by gender, age group, and chronic health conditions across districts in Korea using the 2010-2017 Community Health Surveys
Source: Epidemiol Health. 2024 Jan 4;46:e2024014. doi: 10.4178/epih.e2024014 (PMC11040218; doi:10.4178/epih.e2024014)
Supplement: Supplementary Material 3. — Regression analysis results for the determinants of unhealthy living rates by gender, age group, and urbanity in the general population [file epih-46-e2024014-Supplementary-3.docx]

Supplementary Material 3. Regression analysis results for the determinants of unhealthy living rates by gender, age group, and urbanity in the general population

|  | Total | | 19-44 years | | 45-64 years | | ≥65 years | |
| --- | --- | --- | --- | --- | --- | --- | --- | --- |
|  | β^a^ | R^2^ | β^b^ | R^2^ | β^b^ | R^2^ | β^b^ | R^2^ |
| MEN | | | | | | | | |
| Metropolitan |  | 0.69 |  | 0.49 |  | 0.69 |  | 0.51 |
| **Community socioeconomic factors** |  | 0.28 |  | 0.28 |  | 0.23 |  | 0.08 |
| Living alone | 0.15 | 0.01 | -0.06 | 0.00 | 0.17 | 0.01 | 0.03 | 0.00 |
| Basic recipients | 0.00 | 0.00 | 0.08 | 0.00 | -0.23 | 0.01 | 0.00 | 0.00 |
| High income | -0.03 | 0.00 | -0.12 | 0.02 | -0.01 | 0.00 | -0.02 | 0.00 |
| High education | **-0.23**** | 0.08 | **-0.18*** | 0.05 | **-0.11*** | 0.03 | -0.07 | 0.03 |
| Inoccupation | 0.09 | 0.00 | 0.26 | 0.02 | 0.15 | 0.01 | - | - |
| Manual jobs | 0.05 | 0.00 | 0.11 | 0.01 | 0.14 | 0.02 | - | - |
| **Community interpersonal factors** |  | 0.01 |  | 0.05 |  | 0.01 |  | 0.06 |
| Religious activity | -0.03 | 0.00 | -0.19 | 0.04 | -0.06 | 0.00 | -0.07 | 0.03 |
| Social activity | -0.06 | 0.01 | -0.08 | 0.02 | -0.05 | 0.01 | -0.06 | 0.03 |
| **Community neighborhood factors** |  | 0.02 |  | 0.05 |  | 0.03 |  | 0.04 |
| Pubs | 0.19 | 0.00 | 0.32 | 0.01 | 0.06 | 0.00 | 0.20 | 0.01 |
| Fast-food stores | -1.66 | 0.01 | -4.06 | 0.02 | -1.30 | 0.00 | -0.82 | 0.00 |
| Cigarette retailers | 1.73 | 0.00 | 5.63 | 0.00 | 13.35 | 0.00 | 7.18 | 0.00 |
| Park area | 0.00 | 0.00 | 0.00 | 0.00 | 0.00 | 0.00 | -0.01 | 0.01 |
| Hospital beds | 0.03 | 0.00 | 0.00 | 0.00 | 0.05 | 0.00 | -0.02 | 0.00 |
| Health checkup | 0.01 | 0.00 | 0.10 | 0.01 | -0.11 | 0.01 | -0.05 | 0.01 |
| Unmet medical need | -0.07 | 0.00 | -0.02 | 0.00 | -0.05 | 0.00 | -0.02 | 0.00 |
| Urban |  | 0.55 |  | 0.50 |  | 0.54 |  | 0.42 |
| **Community socioeconomic factors** |  | 0.10 |  | 0.13 |  | 0.05 |  | 0.04 |
| Living alone | -0.11 | 0.00 | -0.20 | 0.03 | 0.06 | 0.00 | -0.06 | 0.01 |
| Basic recipients | 0.33 | 0.01 | -0.10 | 0.00 | -0.11 | 0.00 | 0.04 | 0.00 |
| High income | -0.03 | 0.00 | -0.08 | 0.01 | 0.02 | 0.00 | 0.06 | 0.02 |
| High education | -0.08 | 0.01 | 0.01 | 0.00 | -0.06 | 0.01 | -0.08 | 0.02 |
| Inoccupation | 0.11 | 0.00 | 0.21 | 0.02 | 0.25 | 0.02 | - | - |
| Manual jobs | 0.16 | 0.02 | 0.15 | 0.03 | 0.10 | 0.01 | - | - |
| **Community interpersonal factors** |  | 0.09 |  | 0.05 |  | 0.05 |  | 0.02 |
| Religious activity | -0.17 | 0.03 | -0.11 | 0.01 | -0.11 | 0.01 | -0.02 | 0.00 |
| Social activity | -0.11 | 0.04 | -0.09 | 0.02 | -0.10 | 0.03 | -0.03 | 0.01 |
| **Community neighborhood factors** |  | 0.25 |  | 0.23 |  | 0.28 |  | 0.27 |
| Pubs | 0.94 | 0.05 | 1.27 | 0.05 | 1.19 | 0.03 | 0.50 | 0.02 |
| Fast-food stores | 2.57 | 0.01 | 4.01 | 0.01 | 6.15 | 0.03 | 3.58 | 0.03 |
| Cigarette retailers | -14.73 | 0.01 | -25.26 | 0.02 | -19.33 | 0.01 | -6.06 | 0.00 |
| Park area | **-0.07**** | 0.11 | **-0.09*** | 0.09 | **-0.08*** | 0.07 | -0.04 | 0.07 |
| Hospital beds | -0.09 | 0.03 | -0.09 | 0.02 | -0.10 | 0.01 | -0.03 | 0.00 |
| Health checkup | 0.01 | 0.00 | 0.03 | 0.00 | -0.19 | 0.03 | -0.06 | 0.02 |
| Unmet medical need | -0.02 | 0.00 | 0.08 | 0.00 | -0.17 | 0.01 | 0.09 | 0.02 |
| Rural |  | 0.36 |  | 0.34 |  | 0.47 |  | 0.27 |
| **Community socioeconomic factors** |  | 0.07 |  | 0.09 |  | 0.15 |  | 0.03 |
| Living alone | 0.12 | 0.01 | 0.04 | 0.00 | -0.09 | 0.00 | 0.03 | 0.00 |
| Basic recipients | -0.53 | 0.03 | -0.45 | 0.03 | -0.67 | 0.04 | 0.14 | 0.01 |
| High income | 0.09 | 0.02 | 0.08 | 0.02 | 0.05 | 0.00 | 0.05 | 0.01 |
| High education | 0.05 | 0.00 | -0.06 | 0.00 | -0.08 | 0.00 | 0.08 | 0.00 |
| Inoccupation | 0.01 | 0.00 | 0.00 | 0.00 | 0.08 | 0.00 | - | - |
| Manual jobs | 0.03 | 0.00 | 0.09 | 0.01 | -0.18 | 0.03 | - | - |
| **Community interpersonal factors** |  | 0.01 |  | 0.02 |  | 0.01 |  | 0.02 |
| Religious activity | 0.06 | 0.00 | -0.08 | 0.01 | 0.06 | 0.00 | 0.02 | 0.00 |
| Social activity | -0.08 | 0.01 | -0.05 | 0.01 | -0.06 | 0.01 | 0.03 | 0.01 |
| **Community neighborhood factors** |  | 0.15 |  | 0.11 |  | 0.15 |  | 0.15 |
| Pubs | 0.90 | 0.02 | 1.06 | 0.01 | 1.08 | 0.01 | -0.04 | 0.00 |
| Fast-food stores | 3.45 | 0.04 | 4.74 | 0.03 | 2.27 | 0.01 | 3.23 | 0.07 |
| Cigarette retailers | 4.04 | 0.00 | 1.89 | 0.00 | 11.74 | 0.02 | 6.21 | 0.01 |
| Park area | 0.00 | 0.00 | -0.01 | 0.00 | -0.03 | 0.01 | 0.02 | 0.01 |
| Hospital beds | -0.05 | 0.01 | -0.03 | 0.00 | -0.10 | 0.02 | -0.03 | 0.01 |
| Health checkup | -0.02 | 0.00 | 0.03 | 0.00 | -0.14 | 0.02 | -0.04 | 0.01 |
| Unmet medical need | 0.05 | 0.00 | 0.12 | 0.01 | -0.09 | 0.00 | -0.05 | 0.00 |
| WOMEN | | | | | | | | |
| Metropolitan |  | 0.74 |  | 0.72 |  | 0.66 |  | 0.36 |
| **Community socioeconomic factors** |  | 0.30 |  | 0.34 |  | 0.33 |  | 0.09 |
| Living alone | 0.06 | 0.01 | 0.05 | 0.02 | 0.06 | 0.01 | 0.02 | 0.01 |
| Basic recipients | -0.02 | 0.00 | -0.09 | 0.00 | -0.01 | 0.00 | 0.04 | 0.01 |
| High income | 0.00 | 0.00 | -0.02 | 0.00 | 0.00 | 0.00 | -0.02 | 0.01 |
| High education | **-0.11**** | 0.09 | **-0.17**** | 0.18 | **-0.06*** | 0.04 | -0.03 | 0.01 |
| Inoccupation | 0.14 | 0.02 | 0.09 | 0.01 | **0.14*** | 0.04 | - | - |
| Manual jobs | 0.03 | 0.00 | -0.01 | 0.00 | 0.03 | 0.02 | - | - |
| **Community interpersonal factors** |  | 0.02 |  | 0.03 |  | 0.07 |  | 0.05 |
| Religious activity | -0.01 | 0.00 | **-0.07*** | 0.03 | 0.01 | 0.00 | -0.02 | 0.02 |
| Social activity | -0.04 | 0.02 | -0.01 | 0.00 | **-0.06**** | 0.07 | -0.03 | 0.04 |
| **Community neighborhood factors** |  | 0.05 |  | 0.06 |  | 0.06 |  | 0.11 |
| Pubs | 0.09 | 0.01 | 0.16 | 0.01 | 0.09 | 0.00 | -0.06 | 0.00 |
| Fast-food stores | -0.38 | 0.00 | -1.42 | 0.01 | 0.32 | 0.00 | -0.69 | 0.01 |
| Cigarette retailers | 4.11 | 0.01 | 6.26 | 0.01 | 4.44 | 0.00 | 9.43 | 0.04 |
| Park area | -0.01 | 0.01 | -0.01 | 0.01 | 0.00 | 0.00 | 0.00 | 0.01 |
| Hospital beds | **-0.04*** | 0.03 | **-0.06*** | 0.03 | -0.03 | 0.01 | -0.04 | 0.05 |
| Health checkup | -0.02 | 0.00 | 0.01 | 0.00 | -0.03 | 0.01 | 0.00 | 0.00 |
| Unmet medical need | -0.01 | 0.00 | 0.03 | 0.00 | -0.06 | 0.02 | -0.02 | 0.01 |
| Urban |  | 0.59 |  | 0.52 |  | 0.58 |  | 0.34 |
| **Community socioeconomic factors** |  | 0.14 |  | 0.12 |  | 0.14 |  | 0.10 |
| Living alone | 0.07 | 0.01 | -0.04 | 0.01 | 0.04 | 0.01 | 0.01 | 0.00 |
| Basic recipients | 0.01 | 0.00 | -0.07 | 0.00 | 0.03 | 0.00 | 0.06 | 0.02 |
| High income | -0.04 | 0.01 | -0.08 | 0.05 | -0.02 | 0.01 | 0.00 | 0.00 |
| High education | **-0.16*** | 0.07 | -0.07 | 0.02 | **-0.10*** | 0.07 | -0.16 | 0.06 |
| Inoccupation | -0.14 | 0.01 | -0.03 | 0.00 | -0.19 | 0.04 | - | - |
| Manual jobs | -0.12 | 0.03 | -0.02 | 0.00 | -0.08 | 0.04 | - | - |
| **Community interpersonal factors** |  | 0.03 |  | 0.05 |  | 0.06 |  | 0.02 |
| Religious activity | -0.01 | 0.00 | -0.01 | 0.00 | 0.02 | 0.00 | 0.00 | 0.00 |
| Social activity | -0.05 | 0.02 | -0.06 | 0.04 | -0.07 | 0.06 | -0.02 | 0.02 |
| **Community neighborhood factors** |  | 0.33 |  | 0.34 |  | 0.33 |  | 0.21 |
| Pubs | 0.36 | 0.03 | 0.65 | 0.04 | 0.54 | 0.05 | 0.07 | 0.00 |
| Fast-food stores | 3.14 | 0.05 | 3.74 | 0.03 | 2.83 | 0.04 | 0.94 | 0.01 |
| Cigarette retailers | -9.82 | 0.03 | -22.63 | 0.06 | -8.95 | 0.01 | 7.75 | 0.03 |
| Park area | **-0.03**** | 0.11 | **-0.05**** | 0.15 | **-0.03*** | 0.08 | -0.02 | 0.05 |
| Hospital beds | -0.03 | 0.01 | -0.03 | 0.01 | -0.02 | 0.00 | -0.04 | 0.04 |
| Health checkup | 0.02 | 0.00 | 0.01 | 0.00 | -0.04 | 0.01 | -0.01 | 0.00 |
| Unmet medical need | -0.08 | 0.02 | -0.04 | 0.01 | -0.11 | 0.05 | -0.03 | 0.01 |
| Rural |  | 0.48 |  | 0.48 |  | 0.57 |  | 0.56 |
| **Community socioeconomic factors** |  | 0.08 |  | 0.08 |  | 0.09 |  | 0.06 |
| Living alone | -0.09 | 0.02 | -0.05 | 0.01 | -0.01 | 0.00 | -0.03 | 0.01 |
| Basic recipients | -0.15 | 0.01 | -0.08 | 0.01 | 0.00 | 0.00 | 0.02 | 0.00 |
| High income | 0.02 | 0.01 | 0.01 | 0.00 | 0.03 | 0.01 | 0.03 | 0.01 |
| High education | -0.10 | 0.03 | -0.06 | 0.03 | -0.06 | 0.01 | 0.34 | 0.02 |
| Inoccupation | -0.08 | 0.02 | -0.05 | 0.01 | -0.13 | 0.02 | - | - |
| Manual jobs | -0.07 | 0.02 | 0.00 | 0.00 | **-0.11*** | 0.05 | - | - |
| **Community interpersonal factors** |  | 0.02 |  | 0.06 |  | 0.03 |  | 0.04 |
| Religious activity | 0.03 | 0.01 | 0.01 | 0.00 | 0.04 | 0.02 | 0.00 | 0.00 |
| Social activity | -0.03 | 0.01 | **-0.06*** | 0.06 | 0.01 | 0.00 | 0.02 | 0.03 |
| **Community neighborhood factors** |  | 0.26 |  | 0.21 |  | 0.30 |  | 0.26 |
| Pubs | 0.38 | 0.02 | 0.84 | 0.04 | 0.53 | 0.02 | -0.02 | 0.00 |
| Fast-food stores | **1.96*** | 0.09 | 1.94 | 0.03 | **2.56**** | 0.09 | **2.00**** | 0.14 |
| Cigarette retailers | 1.67 | 0.00 | 2.72 | 0.00 | 1.85 | 0.00 | 0.97 | 0.00 |
| Park area | 0.01 | 0.01 | 0.00 | 0.00 | 0.00 | 0.00 | 0.01 | 0.01 |
| Hospital beds | -0.02 | 0.01 | -0.04 | 0.02 | -0.04 | 0.02 | -0.01 | 0.01 |
| Health checkup | 0.02 | 0.00 | -0.01 | 0.00 | 0.03 | 0.01 | 0.02 | 0.01 |
| Unmet medical need | 0.05 | 0.01 | 0.03 | 0.01 | 0.04 | 0.01 | -0.03 | 0.01 |

β, regression coefficient; R^2^, R-squared value.

^a^ estimated from regression models including age-standardized rates of unhealthy living as dependent variable and age-standardized rates of covariates (except for the number of pubs, fast-food stores, cigarette retailers, and hospital beds per 1,000 people, and park area per person, using crude estimates) as independent variables.

^b^ estimated from regression models including age-specific crude rates of unhealthy living as dependent variable and age-specific crude rates of covariates (except for the number of pubs, fast-food stores, cigarette retailers, and hospital beds per 1,000 people, and park area per person, using crude rates of all age groups) as independent variables.

* p <0.01, ** p<0.001.
