# Supplementary material for: Determinants of unhealthy living by gender, age group, and chronic health conditions across districts in Korea using the 2010-2017 Community Health Surveys
Source: Epidemiol Health. 2024 Jan 4;46:e2024014. doi: 10.4178/epih.e2024014 (PMC11040218; doi:10.4178/epih.e2024014)
Supplement: Supplementary Material 4. — Regression analysis results for the determinants of individual unhealthy lifestyle practice rates by gender in the subgroups of chronic health conditions [file epih-46-e2024014-Supplementary-4.docx]

Supplementary Material 4. Regression analysis results for the determinants of individual unhealthy lifestyle practice rates by gender in the subgroups of chronic health conditions

| Variables | Obesity | | Physical inactivity | | Smoking | | High-risk alcohol consumption | |
| --- | --- | --- | --- | --- | --- | --- | --- | --- |
|  | Risk score ≥1 | Risk score =2 | Risk score ≥1 | Risk score =2 | Risk score ≥1 | Risk score =2 | Risk score ≥1 | Risk score =2 |
|  | β^a^ | β^a^ | β^a^ | β^a^ | β^a^ | β^a^ | β^a^ | β^a^ |
| Depression, men |  |  |  |  |  |  |  |  |
| Living alone | -0.04 | 0.00 | **0.13**^*^ | **0.14**^*^ | -0.01 | 0.00 | 0.01 | -0.05 |
| Basic recipients | 0.00 | -0.01 | -0.02 | 0.02 | 0.03 | 0.02 | 0.06 | 0.00 |
| High income | 0.02 | -0.01 | 0.07 | 0.00 | -0.04 | **-0.07**^**^ | **0.10**^**^ | **0.06**^*^ |
| High education | 0.03 | -0.03 | 0.07 | 0.00 | **-0.12**^**^ | **-0.08**^*^ | 0.05 | -0.01 |
| Inoccupation | 0.04 | 0.02 | 0.02 | 0.02 | 0.10 | 0.09 | 0.02 | 0.08 |
| Manual jobs | 0.06 | -0.01 | 0.01 | 0.04 | 0.05 | **0.07**^*^ | 0.09 | **0.08**^*^ |
| Religious activity | 0.07 | 0.01 | -0.01 | -0.04 | -0.03 | -0.03 | 0.02 | 0.01 |
| Social activity | -0.03 | -0.01 | -0.03 | **-0.09**^*^ | -0.02 | -0.02 | 0.04 | 0.03 |
| Pubs | 0.10 | -0.23 | **0.90**^*^ | 0.55 | -0.31 | -0.34 | 0.53 | 0.32 |
| Fast-food stores | **2.95**^*^ | **1.96**^**^ | 1.68 | 0.68 | **4.73**^**^ | 2.29 | **4.96**^**^ | **3.88**^**^ |
| Cigarette retailers | -8.63 | 1.99 | **-22.64**^*^ | -13.42 | -8.86 | -1.34 | -8.80 | -8.98 |
| Park area | 0.00 | 0.00 | -0.02 | -0.02 | -0.01 | 0.00 | -0.01 | -0.01 |
| Hospital beds | -0.04 | -0.03 | 0.01 | 0.05 | **0.08**^*^ | 0.04 | -0.02 | -0.07 |
| Health checkup | -0.02 | 0.01 | -0.04 | 0.00 | 0.06 | 0.02 | -0.03 | 0.01 |
| Unmet medical need | 0.00 | -0.01 | 0.00 | -0.02 | 0.03 | 0.03 | 0.04 | 0.01 |
| **R-squared** | **0.07** | **0.08** | **0.15** | **0.11** | **0.16** | **0.18** | **0.21** | **0.16** |
| Depression, women |  |  |  |  |  |  |  |  |
| Living alone | -0.04 | 0.00 | 0.10 | **0.13**^*^ | **0.04**^*^ | **0.03**^*^ | 0.02 | 0.02 |
| Basic recipients | 0.01 | -0.02 | -0.06 | -0.11 | -0.04 | -0.01 | -0.07 | -0.04 |
| High income | **-0.04**^*^ | **-0.02**^*^ | 0.06 | 0.05 | 0.02 | 0.01 | 0.03 | 0.01 |
| High education | **-0.12**^**^ | **-0.04**^**^ | 0.06 | 0.00 | **-0.03**^*^ | **-0.03**^*^ | **-0.07**^*^ | -0.03 |
| Inoccupation | 0.00 | -0.01 | 0.07 | 0.08 | **0.05**^*^ | **0.04**^*^ | 0.02 | 0.04 |
| Manual jobs | 0.01 | 0.00 | 0.01 | 0.02 | 0.02 | 0.01 | 0.03 | 0.02 |
| Religious activity | 0.01 | 0.00 | 0.08 | 0.07 | 0.01 | 0.00 | 0.00 | -0.01 |
| Social activity | -0.04 | 0.01 | 0.04 | 0.04 | 0.00 | 0.00 | 0.03 | 0.01 |
| Pubs | -0.16 | -0.14 | **0.78**^*^ | **0.84**^*^ | **0.29**^**^ | **0.24**^**^ | **0.65**^**^ | **0.52**^**^ |
| Fast-food stores | **2.34**^*^ | 0.73 | 0.39 | 0.97 | 0.82 | 0.49 | **2.63**^**^ | 1.00 |
| Cigarette retailers | 2.03 | 2.19 | -4.26 | -7.05 | -4.15 | -1.41 | -7.09 | -2.36 |
| Park area | 0.00 | 0.00 | **-0.04**^*^ | **-0.04**^*^ | -0.01 | -0.01 | -0.01 | -0.01 |
| Hospital beds | **-0.06**^*^ | **-0.03**^*^ | -0.05 | 0.01 | -0.02 | **-0.03**^**^ | 0.00 | -0.04 |
| Health checkup | **0.06**^*^ | 0.01 | -0.06 | -0.02 | 0.00 | 0.00 | -0.01 | -0.01 |
| Unmet medical need | 0.02 | 0.01 | 0.07 | 0.06 | 0.01 | 0.01 | 0.01 | 0.02 |
| **R-squared** | **0.25** | **0.19** | **0.26** | **0.21** | **0.20** | **0.18** | **0.19** | **0.17** |
| Self-rated poor health, men |  |  |  |  |  |  |  |  |
| Living alone | -0.02 | -0.02 | 0.05 | 0.07 | 0.00 | -0.01 | 0.04 | 0.01 |
| Basic recipients | 0.01 | 0.02 | -0.13 | -0.17 | 0.05 | -0.03 | -0.01 | -0.03 |
| High income | **0.07**^**^ | 0.03 | 0.03 | -0.03 | 0.03 | -0.01 | **0.13**^**^ | **0.06**^**^ |
| High education | **0.09**^**^ | 0.03 | 0.10 | 0.04 | **-0.14**^**^ | **-0.12**^**^ | 0.07 | 0.02 |
| Inoccupation | 0.04 | 0.04 | 0.10 | 0.08 | 0.02 | 0.08 | -0.04 | -0.01 |
| Manual jobs | 0.04 | 0.01 | **-0.12**^*^ | -0.09 | 0.04 | **0.07**^*^ | -0.03 | -0.03 |
| Religious activity | -0.01 | 0.01 | 0.09 | 0.07 | **-0.10**^*^ | **-0.09**^*^ | **-0.16**^**^ | **-0.07**^*^ |
| Social activity | -0.03 | -0.02 | 0.05 | 0.02 | -0.03 | 0.00 | 0.03 | 0.04 |
| Pubs | 0.04 | -0.07 | **0.79**^*^ | **0.91**^*^ | 0.33 | 0.02 | **0.67**^*^ | **0.45**^*^ |
| Fast-food stores | **2.47**^*^ | 0.29 | 1.98 | 1.41 | **3.28**^**^ | **2.61**^**^ | **4.48**^**^ | **2.74**^**^ |
| Cigarette retailers | -5.04 | 2.40 | -14.31 | -6.17 | -4.45 | -0.61 | -8.66 | -7.30 |
| Park area | 0.00 | 0.00 | **-0.04**^*^ | -0.02 | 0.00 | 0.00 | **-0.04**^**^ | **-0.03**^**^ |
| Hospital beds | -0.06 | **-0.05**^**^ | -0.04 | -0.02 | -0.01 | -0.01 | **-0.08**^*^ | **-0.05**^*^ |
| Health checkup | 0.01 | 0.01 | 0.03 | -0.01 | 0.05 | 0.03 | 0.01 | 0.00 |
| Unmet medical need | -0.02 | -0.03 | 0.05 | 0.01 | 0.05 | 0.06 | 0.04 | 0.02 |
| **R-squared** | **0.16** | **0.09** | **0.24** | **0.11** | **0.22** | **0.26** | **0.34** | **0.24** |
| Self-rated poor health, women |  |  |  |  |  |  |  |  |
| Living alone | 0.01 | -0.01 | 0.08 | 0.03 | **0.08**^**^ | **0.06**^**^ | **0.11**^**^ | **0.05**^*^ |
| Basic recipients | 0.04 | 0.00 | 0.19 | 0.24 | -0.01 | -0.02 | 0.01 | 0.01 |
| High income | **-0.04**^*^ | -0.02 | **0.09**^*^ | 0.06 | 0.00 | 0.00 | -0.01 | 0.00 |
| High education | **-0.11**^**^ | -0.03 | 0.03 | 0.01 | 0.00 | 0.00 | -0.05 | -0.03 |
| Inoccupation | 0.01 | 0.01 | 0.06 | 0.09 | 0.00 | 0.02 | -0.05 | -0.04 |
| Manual jobs | 0.04 | 0.00 | -0.04 | -0.03 | 0.02 | 0.02 | -0.03 | -0.01 |
| Religious activity | 0.03 | 0.03 | **0.11**^*^ | 0.11 | 0.00 | -0.01 | 0.00 | -0.01 |
| Social activity | 0.01 | 0.00 | 0.06 | 0.06 | -0.02 | -0.02 | 0.02 | 0.01 |
| Pubs | -0.07 | -0.11 | **0.92**^*^ | **0.99**^*^ | **0.27**^*^ | **0.20**^*^ | **0.64**^**^ | **0.30**^*^ |
| Fast-food stores | **3.98**^**^ | 1.02 | 3.57 | **4.09**^*^ | 0.87 | 0.64 | **3.33**^**^ | 0.98 |
| Cigarette retailers | -2.27 | 3.62 | **-21.34**^*^ | **-19.63**^*^ | -5.13 | **-4.84**^*^ | **-17.97**^**^ | **-8.82**^**^ |
| Park area | -0.01 | -0.01 | -0.03 | -0.03 | **-0.01**^*^ | **-0.01**^*^ | -0.02 | -0.01 |
| Hospital beds | -0.05 | 0.00 | **-0.10**^*^ | -0.10 | 0.00 | 0.01 | -0.01 | -0.01 |
| Health checkup | 0.01 | 0.02 | 0.01 | 0.04 | -0.01 | 0.00 | 0.04 | 0.01 |
| Unmet medical need | -0.01 | -0.01 | 0.02 | -0.03 | -0.01 | -0.01 | 0.01 | -0.01 |
| **R-squared** | **0.21** | **0.10** | **0.30** | **0.22** | **0.19** | **0.20** | **0.24** | **0.14** |
| Hypertension, men |  |  |  |  |  |  |  |  |
| Living alone | 0.06 | 0.04 | 0.02 | 0.04 | 0.07 | 0.03 | 0.05 | -0.02 |
| Basic recipients | -0.02 | -0.07 | -0.11 | -0.21 | -0.03 | 0.02 | -0.16 | -0.19 |
| High income | 0.04 | -0.01 | **0.07**^*^ | 0.02 | -0.03 | **-0.04**^*^ | **0.10**^**^ | 0.01 |
| High education | 0.00 | 0.02 | 0.05 | -0.07 | **-0.09**^**^ | **-0.08**^**^ | 0.00 | -0.04 |
| Inoccupation | 0.09 | 0.00 | 0.13 | 0.19 | 0.03 | 0.04 | 0.01 | 0.00 |
| Manual jobs | 0.02 | 0.02 | 0.00 | 0.02 | **0.08**^*^ | **0.08**^**^ | 0.02 | 0.04 |
| Religious activity | 0.03 | 0.00 | 0.01 | 0.01 | -0.05 | **-0.08**^**^ | -0.09 | -0.03 |
| Social activity | -0.01 | -0.02 | -0.04 | -0.05 | 0.01 | 0.00 | 0.00 | -0.02 |
| Pubs | 0.24 | 0.07 | **0.79**^**^ | 0.40 | -0.10 | 0.02 | **0.63**^**^ | **0.52**^**^ |
| Fast-food stores | **1.95**^*^ | 0.43 | -0.55 | 0.26 | **1.96**^*^ | 0.99 | **3.01**^*^ | 1.00 |
| Cigarette retailers | **-13.28**^**^ | -0.94 | -7.84 | -1.20 | 1.96 | -0.78 | -5.61 | -3.21 |
| Park area | 0.00 | 0.01 | -0.01 | -0.01 | -0.01 | -0.01 | -0.02 | -0.01 |
| Hospital beds | **-0.05**^*^ | 0.01 | -0.03 | -0.01 | -0.01 | 0.01 | **-0.07**^*^ | -0.04 |
| Health checkup | 0.00 | 0.00 | 0.01 | 0.06 | **0.07**^*^ | 0.04 | **0.10**^*^ | 0.06 |
| Unmet medical need | -0.03 | -0.04 | 0.00 | -0.06 | 0.01 | -0.01 | 0.07 | 0.03 |
| **R-squared** | **0.13** | **0.07** | **0.13** | **0.06** | **0.25** | **0.34** | **0.29** | **0.15** |
| Hypertension, women |  |  |  |  |  |  |  |  |
| Living alone | 0.03 | 0.01 | **0.25**^*^ | 0.18 | 0.03 | 0.03 | **0.13**^*^ | 0.03 |
| Basic recipients | -0.08 | -0.05 | -0.25 | -0.13 | -0.01 | -0.01 | -0.14 | -0.07 |
| High income | -0.06 | 0.00 | 0.06 | -0.01 | 0.03 | 0.01 | 0.03 | 0.00 |
| High education | **-0.16**^**^ | **-0.07**^*^ | -0.08 | -0.08 | -0.04 | -0.03 | **-0.11**^**^ | **-0.06**^*^ |
| Inoccupation | -0.15 | -0.04 | -0.24 | -0.25 | -0.01 | -0.04 | -0.03 | -0.03 |
| Manual jobs | -0.01 | **-0.06**^*^ | -0.06 | -0.08 | -0.03 | -0.01 | -0.06 | -0.03 |
| Religious activity | 0.05 | 0.03 | **0.14**^*^ | **0.16**^*^ | -0.01 | 0.00 | -0.01 | -0.01 |
| Social activity | 0.01 | 0.01 | -0.01 | -0.05 | 0.00 | -0.01 | **0.08**^**^ | **0.05**^*^ |
| Pubs | 0.02 | -0.10 | **0.93**^*^ | 0.75 | -0.05 | -0.06 | **0.59**^**^ | 0.18 |
| Fast-food stores | **5.81**^**^ | **1.64**^*^ | 3.96 | **4.51**^*^ | **1.49**^**^ | **0.94**^*^ | 1.12 | 1.18 |
| Cigarette retailers | -1.18 | 5.81 | -16.36 | -19.52 | 2.59 | 1.67 | -7.46 | -2.07 |
| Park area | 0.00 | 0.00 | **-0.06**^**^ | -0.04 | 0.00 | 0.00 | -0.01 | -0.01 |
| Hospital beds | -0.02 | 0.01 | -0.07 | -0.08 | -0.02 | -0.01 | -0.03 | 0.00 |
| Health checkup | -0.03 | -0.02 | 0.13 | 0.13 | 0.01 | 0.01 | **0.10**^*^ | **0.05**^*^ |
| Unmet medical need | 0.08 | 0.03 | -0.06 | -0.07 | 0.01 | 0.01 | **0.12**^*^ | 0.03 |
| **R-squared** | **0.15** | **0.07** | **0.21** | **0.14** | **0.09** | **0.05** | **0.21** | **0.13** |
| Diabetes, men |  |  |  |  |  |  |  |  |
| Living alone | -0.08 | -0.01 | 0.04 | 0.08 | -0.03 | -0.04 | 0.01 | -0.03 |
| Basic recipients | 0.14 | **0.15**^*^ | -0.08 | -0.22 | 0.00 | 0.09 | 0.00 | -0.14 |
| High income | **0.08**^*^ | **0.05**^*^ | **0.15**^**^ | **0.10**^*^ | 0.04 | 0.00 | **0.11**^**^ | 0.05 |
| High education | 0.08 | 0.03 | 0.09 | -0.04 | **-0.08**^*^ | **-0.08**^**^ | 0.07 | 0.04 |
| Inoccupation | 0.09 | 0.07 | 0.20 | 0.22 | 0.11 | 0.03 | 0.11 | 0.04 |
| Manual jobs | 0.04 | 0.04 | -0.02 | 0.03 | **0.09**^*^ | **0.09**^**^ | 0.02 | 0.06 |
| Religious activity | 0.01 | 0.03 | 0.06 | 0.02 | -0.09 | **-0.09**^*^ | -0.03 | -0.02 |
| Social activity | 0.03 | 0.02 | 0.05 | 0.01 | -0.02 | 0.00 | 0.04 | 0.04 |
| Pubs | 0.11 | -0.10 | **0.71**^*^ | 0.51 | 0.22 | 0.06 | 0.42 | 0.17 |
| Fast-food stores | **3.81**^*^ | 0.93 | 0.82 | 1.46 | 2.31 | 1.53 | 2.76 | 1.42 |
| Cigarette retailers | **-15.84**^*^ | 0.41 | -11.45 | 1.53 | -11.50 | -5.69 | -3.89 | 4.05 |
| Park area | -0.01 | 0.01 | -0.03 | -0.02 | 0.00 | 0.01 | -0.02 | -0.02 |
| Hospital beds | -0.05 | 0.00 | 0.02 | 0.04 | 0.04 | 0.03 | 0.00 | 0.01 |
| Health checkup | 0.08 | 0.00 | 0.05 | 0.07 | 0.05 | **0.07**^*^ | 0.04 | 0.01 |
| Unmet medical need | 0.01 | -0.04 | 0.02 | -0.03 | -0.06 | -0.01 | -0.08 | -0.02 |
| **R-squared** | **0.19** | **0.10** | **0.24** | **0.11** | **0.14** | **0.18** | **0.17** | **0.08** |
| Diabetes, women |  |  |  |  |  |  |  |  |
| Living alone | 0.04 | 0.00 | 0.14 | 0.09 | 0.04 | 0.02 | -0.03 | 0.00 |
| Basic recipients | **0.29**^**^ | 0.05 | -0.04 | -0.07 | 0.04 | 0.03 | 0.02 | 0.02 |
| High income | -0.01 | 0.01 | 0.09 | 0.06 | **0.03**^*^ | **0.02**^*^ | 0.01 | 0.00 |
| High education | -0.12 | -0.06 | -0.02 | -0.07 | **-0.05**^**^ | **-0.06**^**^ | -0.03 | **-0.05**^*^ |
| Inoccupation | -0.06 | -0.10 | -0.01 | 0.11 | -0.03 | -0.01 | 0.01 | -0.05 |
| Manual jobs | 0.05 | 0.02 | -0.06 | -0.10 | 0.00 | 0.00 | 0.00 | 0.01 |
| Religious activity | 0.08 | 0.02 | 0.12 | 0.11 | 0.02 | 0.02 | 0.04 | **0.05**^**^ |
| Social activity | -0.06 | -0.03 | -0.01 | 0.03 | **-0.04**^**^ | -0.02 | 0.01 | -0.03 |
| Pubs | -0.14 | -0.10 | 0.80 | **0.98**^*^ | -0.02 | 0.03 | 0.33 | 0.22 |
| Fast-food stores | **5.40**^**^ | 1.53 | **5.17**^*^ | **4.86**^*^ | **2.12**^**^ | **1.87**^**^ | 1.68 | 0.69 |
| Cigarette retailers | -13.17 | -1.59 | -17.85 | -12.29 | -0.45 | -0.55 | 4.52 | -3.48 |
| Park area | 0.00 | -0.01 | -0.04 | -0.03 | 0.00 | 0.00 | -0.01 | 0.00 |
| Hospital beds | -0.07 | -0.03 | **-0.15**^**^ | **-0.13**^*^ | -0.02 | -0.02 | -0.05 | -0.01 |
| Health checkup | 0.01 | 0.02 | 0.05 | 0.09 | -0.02 | -0.02 | 0.01 | 0.03 |
| Unmet medical need | 0.00 | 0.07 | -0.07 | -0.03 | -0.03 | -0.02 | -0.05 | -0.01 |
| **R-squared** | **0.13** | **0.09** | **0.18** | **0.16** | **0.17** | **0.19** | **0.07** | **0.11** |
| Arthritis, men |  |  |  |  |  |  |  |  |
| Living alone | -0.02 | -0.01 | -0.04 | -0.02 | -0.03 | -0.02 | 0.00 | 0.00 |
| Basic recipients | -0.02 | 0.00 | -0.08 | -0.05 | 0.04 | -0.01 | 0.00 | -0.01 |
| High income | -0.02 | 0.01 | 0.00 | -0.02 | **-0.06**^**^ | **-0.04**^*^ | **0.05**^*^ | 0.02 |
| High education | 0.02 | 0.00 | 0.01 | -0.07 | **-0.09**^**^ | **-0.09**^**^ | -0.03 | -0.02 |
| Inoccupation | 0.02 | **0.04**^**^ | 0.03 | 0.06 | 0.05 | 0.04 | 0.02 | 0.03 |
| Manual jobs | -0.03 | 0.01 | **-0.11**^**^ | -0.08 | 0.06 | **0.07**^**^ | -0.03 | 0.00 |
| Religious activity | 0.06 | 0.00 | **0.11**^*^ | **0.14**^**^ | **-0.08**^**^ | -0.04 | -0.03 | -0.01 |
| Social activity | 0.01 | 0.01 | -0.01 | -0.03 | 0.02 | 0.03 | 0.01 | 0.01 |
| Pubs | -0.10 | 0.07 | **0.63**^**^ | **0.51**^**^ | -0.17 | -0.10 | **0.27**^*^ | 0.08 |
| Fast-food stores | 0.85 | -0.09 | -0.46 | 0.35 | 0.73 | 0.05 | **1.80**^**^ | **1.53**^**^ |
| Cigarette retailers | -3.53 | -1.19 | -2.40 | -1.41 | 0.51 | 2.13 | -0.02 | -1.90 |
| Park area | 0.00 | 0.00 | -0.01 | 0.00 | 0.00 | 0.00 | -0.01 | 0.00 |
| Hospital beds | -0.02 | 0.00 | -0.01 | 0.00 | -0.01 | -0.01 | -0.03 | -0.02 |
| Health checkup | 0.01 | 0.01 | 0.00 | -0.01 | 0.00 | -0.01 | 0.02 | 0.01 |
| Unmet medical need | -0.05 | -0.02 | -0.03 | -0.02 | **0.09**^**^ | **0.11**^**^ | 0.00 | 0.02 |
| **R-squared** | **0.07** | **0.09** | **0.19** | **0.14** | **0.21** | **0.24** | **0.13** | **0.07** |
| Arthritis, women |  |  |  |  |  |  |  |  |
| Living alone | 0.01 | 0.01 | 0.10 | 0.07 | 0.02 | **0.02**^*^ | 0.04 | 0.01 |
| Basic recipients | 0.03 | 0.00 | 0.05 | 0.08 | -0.01 | 0.00 | -0.01 | 0.02 |
| High income | -0.02 | 0.00 | 0.02 | -0.03 | 0.01 | 0.00 | 0.00 | 0.00 |
| High education | -0.03 | 0.00 | -0.02 | -0.06 | -0.01 | -0.01 | -0.01 | -0.02 |
| Inoccupation | 0.11 | 0.02 | -0.11 | -0.08 | 0.02 | 0.01 | **0.08**^*^ | **0.03**^*^ |
| Manual jobs | 0.04 | 0.00 | -0.11 | -0.12 | 0.00 | 0.00 | 0.00 | 0.00 |
| Religious activity | **0.05**^*^ | 0.01 | **0.19**^**^ | **0.20**^**^ | 0.01 | **0.01**^*^ | 0.02 | 0.01 |
| Social activity | **-0.05**^*^ | -0.01 | -0.03 | -0.05 | -0.01 | -0.01 | **-0.03**^*^ | -0.01 |
| Pubs | -0.11 | -0.05 | **0.41**^*^ | **0.40**^*^ | 0.01 | 0.00 | **0.15**^**^ | **0.06**^**^ |
| Fast-food stores | **1.49**^**^ | **0.47**^**^ | 1.09 | 1.04 | **0.36**^**^ | **0.30**^**^ | **0.44**^*^ | 0.12 |
| Cigarette retailers | 1.08 | -0.42 | -7.00 | -4.42 | 0.11 | 0.33 | -1.01 | -0.24 |
| Park area | 0.00 | 0.00 | -0.01 | -0.01 | 0.00 | 0.00 | 0.00 | 0.00 |
| Hospital beds | **-0.03**^**^ | **-0.01**^**^ | -0.03 | -0.03 | 0.00 | 0.00 | -0.01 | 0.00 |
| Health checkup | -0.06 | -0.01 | -0.13 | -0.14 | **-0.03**^*^ | -0.02 | -0.01 | -0.01 |
| Unmet medical need | **-0.08**^**^ | 0.00 | **-0.18**^**^ | **-0.21**^**^ | -0.01 | 0.00 | **-0.03**^*^ | -0.01 |
| **R-squared** | **0.21** | **0.13** | **0.26** | **0.24** | **0.13** | **0.14** | **0.23** | **0.16** |

^a^ estimated from regression models including age-standardized rates of individual unhealthy lifestyle practices as dependent variable and age-standardized rates of covariates (except for the number of pubs, fast-food stores, cigarette retailers, and hospital beds per 1,000 people, and park area per person, using crude estimates) as independent variables.

* p <0.01, ** p<0.001.
